# Physiological, genetic and inflammatory factors influencing somatic cell count in goat milk: a review

**DOI:** 10.1186/s13028-026-00880-7

**Published:** 2026-10-01

**Authors:** Jovita Einorytė, Giedrius Palubinskas, Birutė Šlyžienė, Renata Baltaduonytė, Evaldas Šlyžius

**Affiliations:** Department of Animal Breeding, Faculty of Animal Science, Lithuanian University of Health Sciences, Tilzes Str. 18, Kaunas, LT-47181 Lithuania

**Keywords:** Dairy goats, Intramammary infection, Mastitis, Milk quality, Multifactorial variability, SCC interpretation

## Abstract

Somatic cell count (SCC) is widely used as an indicator of milk quality and udder health in ruminants; however, its interpretation in goats remains complex due to species-specific physiology and multifactorial variability. A key challenge in goat dairy production is distinguishing physiological from pathological SCC elevation. This critical narrative review synthesizes current knowledge on physiological, genetic, environmental, and inflammatory determinants of SCC in goat milk and evaluates their implications for milk quality and udder health assessment. The evidence indicates that SCC in goats is inherently elevated due to the apocrine milk secretion mechanism and is further influenced by factors such as lactation stage, parity, environmental conditions, and management practices. Genetic effects are moderate and context-dependent, while intramammary infection remains the primary pathological driver of SCC increase. These findings demonstrate that SCC cannot be reliably interpreted using fixed threshold values. Instead, its diagnostic value depends on context-dependent evaluation. Based on the reviewed evidence, an integrated framework for SCC interpretation is proposed, enabling differentiation between physiological and pathological SCC elevation. This approach supports more accurate diagnostic decision-making and improved milk quality management in goat production systems.

## Background

Over the past few decades, goat milk production has become an increasingly important segment of global agriculture, particularly in developing countries where goats represent a vital source of food security and income. In developed regions, goat milk consumption is also expanding due to its distinctive nutritional profile and recognized health benefits. Compared with cow milk, goat milk contains lower lactose levels, higher concentrations of easily digestible fatty acids, and a β-casein-to-αs1-casein ratio (70%/30%) that is closer to that of human milk. These characteristics enhance its suitability for individuals with lactose intolerance or sensitivity to cow milk proteins and contribute to its growing nutritional and economic relevance [1–3].

Milk quality assessment in goats extends beyond nutritional composition and includes SCC, a key indicator of udder health and milk safety. However, interpretation of SCC in goat milk is considerably more complex than in cows. Due to the apocrine mechanism of milk secretion, goat milk naturally contains higher baseline SCC levels. At the same time, SCC may increase due to pathological conditions such as mastitis or uterine inflammation, as well as non-pathological physiological factors including parity, lactation stage, hormonal changes, stress, nutrition, environmental conditions, and farm management practices [4].

Regulatory SCC thresholds for goat milk vary between countries, ranging from 500,000 to 1,000,000 cells/mL. These differences reflect ongoing scientific discussion regarding the diagnostic and technological significance of elevated SCC. Physiological increases—particularly during late lactation or seasonal changes—may not compromise milk safety, whereas inflammation-associated SCC elevation may involve pathogenic microorganisms and toxins, negatively affecting milk quality, safety, and technological properties. This variability is discussed in the context of EU and US benchmarks, including EU proposals around 1,500,000 cells/mL and the commonly cited US limit of 1,000,000 cells/mL for caprine milk [5]. This variability creates uncertainty in herd health management, milk quality control, and regulatory decision-making.

One of the principal challenges in goat dairy production is distinguishing physiological from pathological SCC elevation, as multiple interacting genetic, environmental, technological, and inflammatory factors influence SCC variability and complicate its diagnostic interpretation.

The research objective of this review is SCC in goat milk and the multifactorial determinants of its variability. Although numerous studies have examined individual determinants of SCC in goats, current knowledge remains fragmented across physiological, genetic, and management-related domains. A comprehensive synthesis integrating these factors into a unified interpretation framework is still lacking.

This review aims to provide an integrated overview of the factors influencing SCC in goat milk. Specifically, it addresses physiological and management-related determinants, genetic and polymorphic influences, inflammatory and infectious causes, the diagnostic value of SCC and electrical conductivity, and current approaches for SCC monitoring and milk quality prediction.

## Methods

The search strategy included combinations of keywords related to goat milk, somatic cell count (SCC), mastitis, and associated factors.

**Fig. 1:**
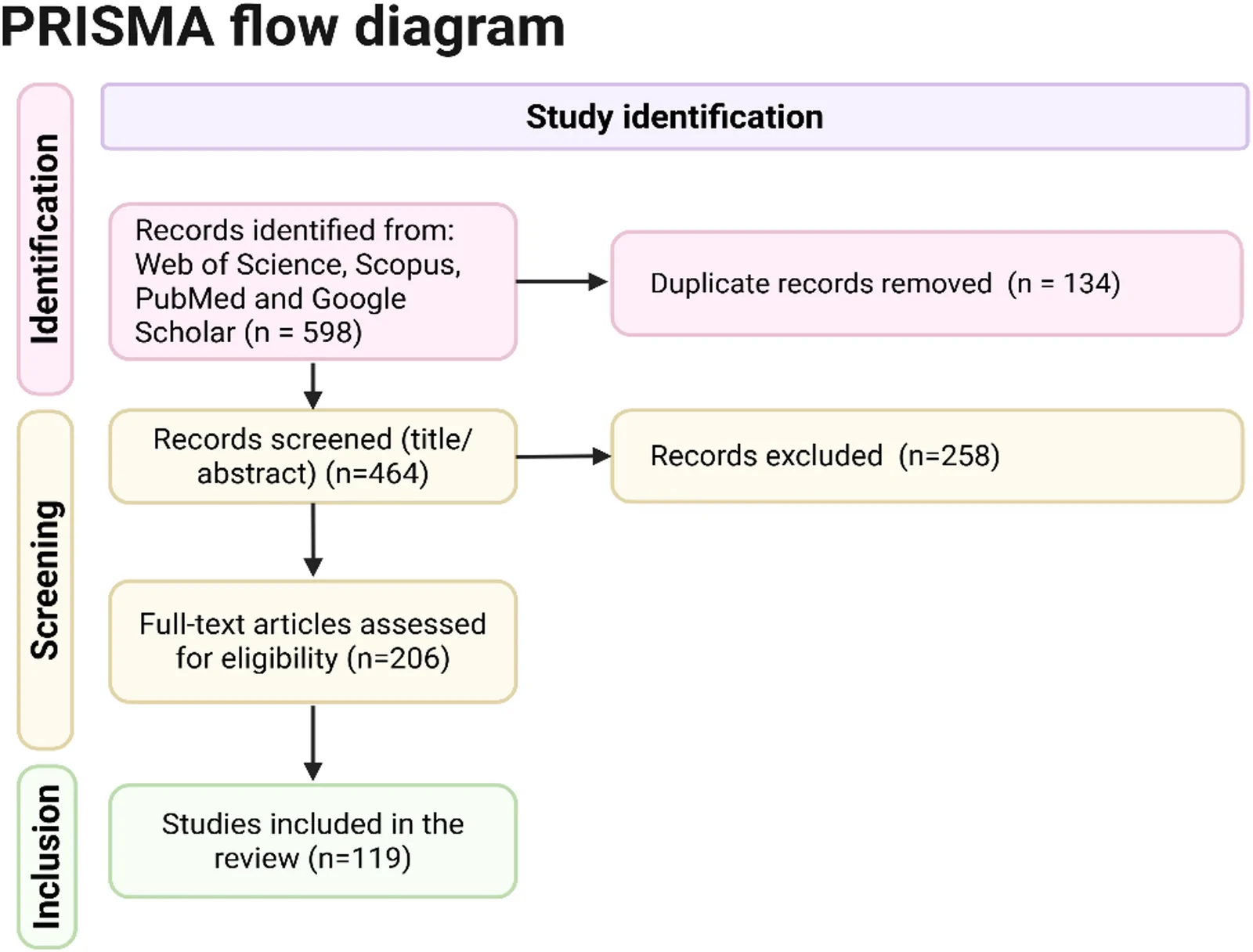
PRISMA flow diagram of literature selection. (Created by the author using BioRender)

A structured narrative literature review was conducted, guided by selected PRISMA reporting elements to enhance transparency, although a full systematic review protocol was not applied (Fig. 1). The review focused on SCC in goat milk and factors influencing its variability, including physiological, genetic, inflammatory, environmental, technological, and management-related determinants.

The literature search covered publications from 2000 to 2025 and was limited to articles published in English. Relevant literature was retrieved from scientific databases, including Web of Science, Scopus, and PubMed, and complemented by searches in Google Scholar to ensure broader coverage of academic sources and grey literature.

The search strategy was based on combinations of keywords using Boolean operators (AND, OR) to refine the results: the Boolean operator OR was used to combine synonymous or conceptually related terms, whereas AND was used to link the main concept of goat milk and SCC with specific thematic determinants. The search combinations included these search phrases (“goat milk” AND (“somatic cell count” OR “SCC”)), (“goat milk” AND (“mastitis” OR “intramammary infection”)), and (“goat milk” AND “SCC” AND (“lactation” OR “parity” OR “udder conformation” OR “genetic polymorphism” OR “feeding”)).

The initial search yielded 598 records, of which 206 were retained after removal of duplicates and title and abstract screening. Following full-text assessment, 119 publications met the inclusion criteria and were included in the final analysis.

Sources were included when they directly addressed SCC in goats or provided relevant comparative physiological, genetic, inflammatory, or technological context. Peer-reviewed original research articles were prioritized, while review papers, book chapters, and other academic sources were included when they contributed essential background or conceptual information. Publications not relevant to goat milk SCC or not aligned with the aims of this review were excluded.

The selected literature was analysed and organised according to thematic relevance and grouped into physiological, genetic, inflammatory, diagnostic, and technological categories to provide an integrated overview of current scientific knowledge on SCC variability in goat milk. This study was conducted as a structured critical narrative review, applying selected PRISMA elements to ensure transparency in literature identification and selection.

## Review

### Physiological factors influencing SCC in goat milk

#### Global standards for goat milk SCC

Milk SCC forms the basis of abnormal milk control programs. In the United States, legal SCC limits are 1,000 × 10³ cells/mL for goats and 750 × 10³ cells/mL for sheep, while the bulk tank limit for goats is 1,500,000 cells/mL. In goats without intramammary infection (IMI), SCC typically ranges from 270 to 2,000 × 10³ cells/mL, exceeding values observed in sheep (10–200 × 10³ cells/mL) and cattle [6, 7].

Neutrophils predominate in uninfected goat milk (45–74% of SCC), whereas macrophages dominate in cows and sheep. This difference is mainly related to the apocrine secretion mechanism, which releases cytoplasmic particles (≈ 150 × 10³/mL) similar in size to somatic cells; therefore, DNA-specific counting methods are required for accurate SCC determination in goats. Although IMI significantly increases SCC, non-infectious factors such as parity, lactation stage, season, and milking practices also contribute. Increased SCC has been associated with reduced milk yield and fat content. Dry-period antibiotic therapy and post-milking teat disinfection reduce IMI incidence and SCC similarly to dairy cattle [6, 7].

Despite the growing economic importance of dairy goat production in Europe [1], the European Union has not established a uniform legal SCC threshold for goat milk, unlike the United States [8]. As production expands, optimizing milk quality parameters remains essential for consumer safety [9]. Although knowledge of goat milk quality parameters continues to evolve, research in this field has increased substantially in recent years [10].

#### Functional anatomy and physiology of the goat mammary gland related to milk secretion

Anatomical characteristics of the goat mammary gland influence milk secretion and SCC compared with other dairy species [11]. The udder consists of two independent glands composed of parenchyma and stroma, supported by medial and lateral suspensory ligaments branching into secondary laminae. Each half contains multiple lobes draining into six to nine milk ducts forming a gland cistern, which empties into the teat cistern [12]. Furstenberg’s ring demarcates the udder–teat junction. The teat wall includes mucosa, vascular connective tissue, circular and longitudinal muscle layers, and epithelium. The streak canal (0.5–1.0 cm), lined with keratin-producing squamous epithelium and controlled by a muscular sphincter, functions as an important anatomical barrier [13–15]. Compared with cows, goats possess a relatively large cisternal storage capacity [13–15].

Milk is synthesized in alveoli and transported to cisterns for storage until milking [11]. In goats, approximately 30% of milk is stored in alveoli and 70% in the cistern, whereas in cows these proportions are reversed. Cistern storage varies between 25% and 70% depending on breed and lactation stage but generally represents at least two-thirds in dairy breeds [16]. Consequently, milk ejection in goats is less dependent on oxytocin-driven let-down than in cows [17]. Udder structure and function change during gestation and lactation under neuroendocrine regulation, influencing milk yield and composition [18, 19]. Thus, milk quality varies with internal factors (breed, lactation stage) and external factors (nutrition, environment) [8, 20].

Due to large cisternal capacity, milk may be partially ejected by passive gravity. Alveolar milk ejection begins with afferent stimulation via sensory receptors in the teat and udder base and may also be triggered by visual, auditory, or olfactory stimuli, developing into a conditioned reflex [12, 15]. The ejecto-lacteal reflex is regulated primarily by oxytocin (OXT) and antidiuretic hormone, transported via the external pudendal artery. Adrenaline inhibits milk let-down by constricting blood vessels and limiting OXT access to myoepithelial cells [12].

Lactation in goats lasts approximately 10 months and peaks between weeks 5 and 10 postpartum [21], compared with about 5 months in sheep (peak weeks 3–4) [22] and 11 months in cows (peak weeks 6–7) [23]. However, lactation length in goats varies depending on breed and nutrition [24].

**Fig. 2:**
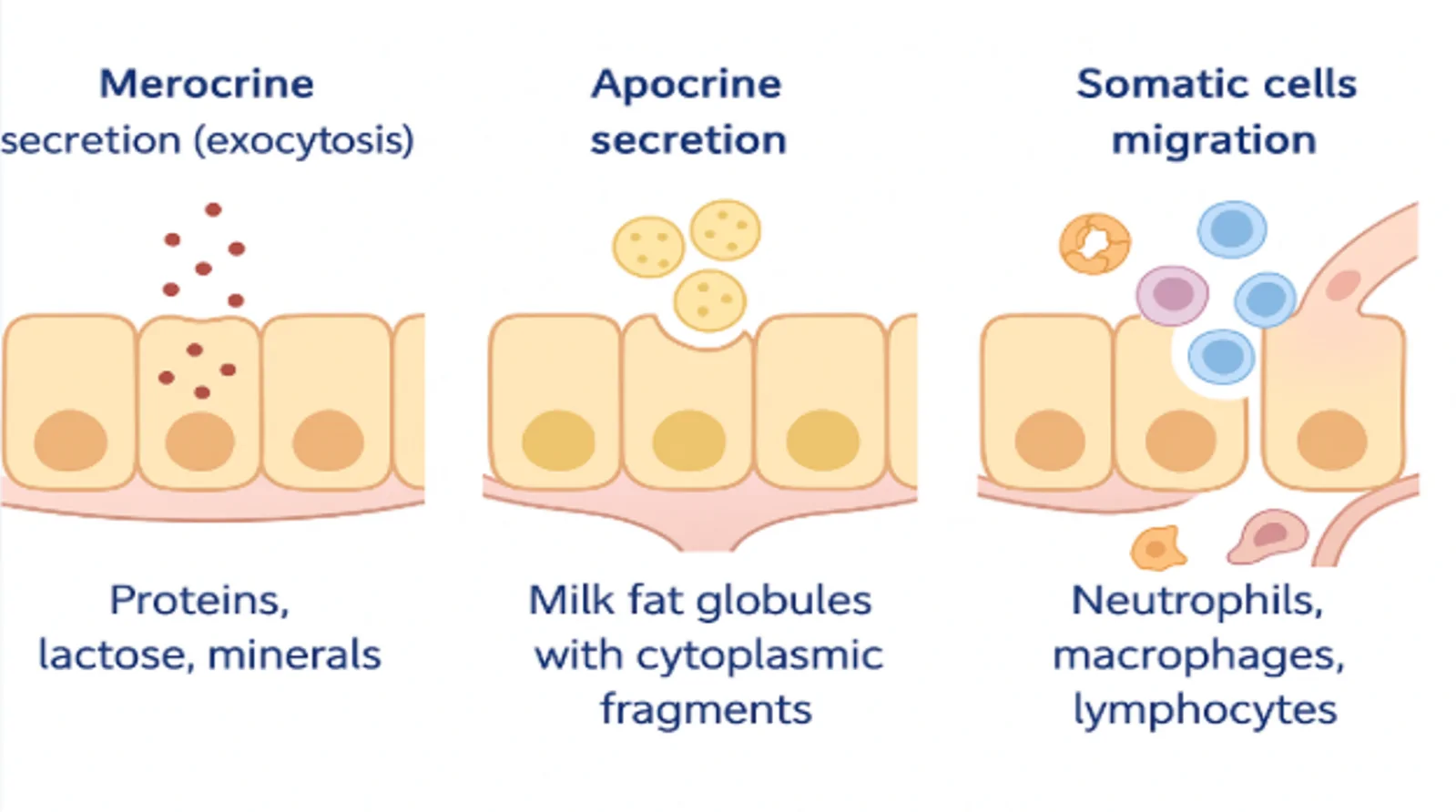
Comparison of merocrine and apocrine secretion pathways in goat milk. (figure created by the author using BioRender; refined with AI-assisted tools). Merocrine secretion (exocytosis) involves the release of proteins, lactose, and minerals into the lumen. In contrast, apocrine secretion is characterized by the discharge of milk fat globules surrounded by cytoplasmic fragments of secretory cells. Somatic cell migration contributes additional components such as neutrophils, macrophages, and lymphocytes. The illustration was created by the authors using BioRender.com, based on descriptions of milk secretion mechanisms reported in the literature [25–29]

Goat milk secretion is primarily apocrine. During this process, cytoplasmic fragments are released into the lumen, while most of the cell structure remains intact. Both apocrine and merocrine pathways contribute to milk composition [25, 26] (Fig. 2).

Cytoplasmic fragments usually lack nuclei and contain proteins, lipids, and micelles [27]. Electron microscopy demonstrates fat globules surrounded by cytoplasmic material and fragments of secretory cells [25, 28]. These fragments have no pathological significance [27] but may be misidentified as somatic cells during SCC determination [26]. Goat milk secretion has even been described as partially holocrine, as some whole cells may enter the lumen [29].

Although sheep milk secretion is also apocrine, cytoplasmic fragment concentration averages about 15 × 10³/mL, markedly lower than approximately 150 × 10³/mL in goats [6]. In contrast, cow mammary secretion is merocrine, with milk components released without cytoplasmic loss [25, 30], explaining species-specific baseline SCC differences [27, 31].

#### Influence of litter size

The number of kids per goat may influence SCC alongside parity and lactation stage. Higher SCC was reported in goats with twins (log10SCC 5.66) than in goats nursing a single kid (log10SCC 5.45) [32], while increased SCC, protein, and lactose content was observed in goats with more offspring [33].

In contrast, higher SCC values (> 500 × 10³ cells/mL) were found in goats suckling fewer kids (mean 1.91) compared with those with more offspring (2.1–2.29) [34]. These inconsistent findings indicate that the relationship between litter size and SCC depends on additional physiological and management factors. These contradictory findings may reflect differences in herd management, lactation stage at sampling, and nutritional status of the animals, all of which may influence SCC independently of litter size.

#### Milk composition

Milk composition in goats is dynamic and influenced by physiological and environmental conditions [26, 35]. Unlike cow milk, goat milk composition varies seasonally and reflects the animal’s physiological status [35]. Toward the end of lactation, protein, fat, and mineral content increase, while lactose decreases [36]. Compared with cow and sheep milk, goat milk differs in fatty acid profile, lactose, enzymes, vitamins, minerals, and notably exhibits higher SCC [37].

Goat milk naturally contains numerous epithelial cells and fragments due to epithelial turnover [26]. Somatic cells consist mainly of epithelial cells and leukocytes (lymphocytes, macrophages, polymorphonuclear neutrophils (PMN)) [31, 38]. In healthy cows SCC is typically ≤ 100,000 cells/mL and around 200,000 cells/mL in sheep [39, 40], whereas in uninfected goats it averages ~ 300,000 cells/mL and may reach ≥ 2,000,000 cells/mL late in lactation [9, 41].

PMN represent the predominant cell type in both healthy (45–74%) and infected (71–86%) goat milk [6]. Macrophages account for 15–41% in uninfected and 8–18% in infected milk, while lymphocytes comprise 9–20% and 5–11%, respectively [42]. Epithelial cells may constitute up to 27% of SCC in uninfected goat milk [9]. These differences in cellular composition should be considered when interpreting SCC in goats.

Somatic cells contribute endogenous enzymes. In goats, PMN are a major source of proteolytic enzymes such as cathepsins and elastase [38, 43]. The principal protease systems include plasmin–plasminogen and cathepsin D (EC 3.4.23.5). Plasmin (EC 3.4.21.7), associated with casein micelles and somatic cells, directly affects technological properties [44]. Elevated SCC enhances proteolytic activity, reduces curd firmness, and alters organoleptic quality [45].

SCC increases during mammary infection and is therefore widely used in the diagnosis of IMI [26, 31]. In subclinical mastitis, the proportion of PMN increases markedly in cows and sheep but less so in goats, where PMN already constitute approximately 45–74% of the somatic cell population under physiological conditions [6, 38]. Beyond immune protection, somatic cells release enzymes that affect milk production and the technological quality of dairy products [8].

Compared with cow milk, goat milk contains higher levels of goat milk oligosaccharides (gMOS), medium-chain triglycerides, and β-casein, and lower αs1-casein [3]. Although a direct association between SCC and gMOS has not been confirmed, their anti-inflammatory properties may indirectly modulate SCC [46].

Mastitis induces oxidative and nitrosative stress, altering milk composition and reducing yield [47]. Increased SCC is accompanied by changes in catalase, lactoferrin oxidase, and glutathione peroxidase activity. Elevated nitric oxide, nitrite, and nitrate levels impair milk quality and technological properties and may serve as indicators of subclinical mastitis [47].

#### Impact of goat milk proteins and SCC on milk production

Goat milk has a distinctive biochemical composition affecting processing suitability. Compared with cow milk, it contains higher oligosaccharides, medium-chain triglycerides, and β-casein, lower αs1-casein, fewer protease peptones, and more non-protein nitrogen [3, 48]. Lactose content is generally lower, mineral composition similar (with slightly higher potassium and chloride), and carotenoids absent; vitamins B6 and B12 are present in small amounts [3, 40, 49].

Casein content is closely linked to SCC. Elevated SCC increases leukocyte-derived proteases, degrading casein and impairing coagulation and cheese yield [50]. Mastitis-related SCC elevation is also associated with increased serum proteins such as albumin, altering protein balance [3, 51].

Some researches showed that αs1-casein levels depend on the CSN1S1 genotype, although cheese yield correlated more strongly with total protein, fat, and casein content [39, 49]. Lactose and pH are negatively correlated with log SCC [52–54], although another results [55] showed no significant differences between SCC groups (< 300 × 10³ vs. <200 × 10³ cells/mL). Higher SCC in August was accompanied by lower lactose and pH without evidence of IMI [56].

Seasonal protein balance can be standardized through microfiltration (MF) and micro- and diafiltration (MDF), improving cheese quality and stabilizing production during lactation [50]. MF supports consistent coagulation, while MDF reduces lactose and eliminates the curd wash step in hard cheese production.

#### Parity, reproductive status and lactation stage

SCC in goat milk is strongly influenced by parity and lactation stage, reflecting structural and functional changes in the mammary gland over time [14]. SCC was reported to increase significantly up to the fifth parity [57], while the proportion of low SCC recordings (≤ 245,000 cells/mL) decreased from 45.2% in first-parity to 20.8% in older goats. Similar trends were found in several studies [58–60], with higher SCC and total bacterial count (TBC) also reported in third-parity goats compared with primiparous animals, possibly reflecting cumulative pathogen exposure and progressive epithelial deterioration [14, 57].

In contrast, decreasing SCC with increasing parity was found [34], as goats in the low SCC group (≤ 470 × 10³ cells/mL) had the highest mean parity (2.91). Higher SCC was reported in third-parity goats (2.16 × 10⁵ cells) compared with first (1.89 × 10⁵) and second (1.87 × 10⁵) parity [61], demonstrating a significant parity × lactation stage interaction [61, 62]. These findings indicate that parity effects are not uniform and may depend on additional physiological or management factors.

Reproductive events also influence SCC independently of mammary infection. A significant estrus-related increase in SCC (mean + 315,000 cells/mL) was demonstrated [57], with the largest increase observed in fourth-parity goats (422,000 cells/mL) and the smallest in second-parity goats (241,000 cells/mL). Likewise, hormonally induced estrus caused a transient 3.5-fold increase in SCC regardless of infection status [63], as also supported by related findings [59, 63]. These physiological elevations should therefore be considered when interpreting SCC as an indicator of IMI [62–64].

Lactation stage is consistently identified as a major determinant of SCC. Several studies demonstrated increasing SCC in late lactation [59, 61] while another study reported higher SCC and TBC toward the end of lactation and lower values immediately after kidding [62]. Although increasing SCC during lactation was observed [64], total leukocyte numbers remained stable due to constant neutrophil proportions. However, substantial individual variability was observed, with no significant differences between the first and fifth lactations in some breeds [37].

While most studies report an increase in SCC during late lactation, a decrease after 300 days in milk was also observed [57]. This finding should not be interpreted as contradicting the general late-lactation trend, as the authors suggested that it may reflect selection of goats with favourable production characteristics or specific indoor management conditions at this advanced stage of lactation. Thus, SCC trajectories during very prolonged lactation may differ from the general pattern observed during conventional late lactation. Lower SCC was also reported in goats giving birth at night compared with daytime parturition [58].

Milk yield generally increases with parity [59], yet a negative association between milk yield and SCC has been reported, with SCC decreasing as yield increased [58]. Another research [34] similarly described negative correlations between milk yield, days in milk, and SCC.

SCC above 2,000 × 10³ cells/mL significantly delayed rennet coagulation time (RCT) and impaired curd formation [65], with lactation stage further affecting milk quality, particularly under mountain grazing conditions. Transcriptomic analysis revealed stage-dependent gene expression: antimicrobial and mucus-related genes predominated in early lactation, whereas genes related to major milk proteins and ABC transporters were upregulated later [66].

Because reproductive activity in dairy goats is largely regulated by photoperiod, kidding and the onset of lactation usually occur within defined seasonal periods. Consequently, seasonal variation and lactation stage are closely interconnected, and seasonal effects on SCC should be interpreted in the context of the reproductive cycle and lactation dynamics.

#### Seasonal impact on goat SCC

Building upon the physiological changes associated with reproduction and lactation, several studies have specifically evaluated season as an independent factor influencing SCC. Although seasonal patterns differ among geographical regions and production systems, season consistently contributes to SCC variability through its interaction with photoperiod, environmental conditions, and kidding distribution.

The highest SCC and TBC were reported in winter (3.95 ± 7.14 × 10⁶ cells/mL; 2.23 ± 5.54 × 10⁴ CFU/mL) and the lowest in spring (0.94 ± 1.54 × 10⁶ cells/mL; 0.22 ± 0.61 × 10⁵ CFU/mL) [67]. In contrast, the highest SCC in autumn and the lowest in winter were observed in another study, with season explaining 13% of SCC variability and influencing fat, lactose, and total solids [54].

Increased SCC during summer and autumn, peaking in October (log10 3.51), with the lowest values in February (log10 3.25), paralleling TBC variation [53]. Similarly, lower log10SCC was reported in spring (5.45) than in summer (5.56) and autumn (5.60) [32]. Monthly effects on SCC, milk yield, composition, freezing point, pH, and microbial count were also demonstrated with SCC increasing from May to July [68]. Likewise, peak SCC was observed in June–July [48], and a 35% increase was reported between May and August [56], and similar seasonal trends were described in other studies [58, 69, 70].

SCC peaks were identified in June (early lactation), July–August (mid-lactation), and August (late lactation) [57]. Similarly long-term increases in SCC and TBC were reported over 22 years, with peak values in January, coinciding with the kidding season in the Netherlands [62]. SCC (6.70 log10 cells/mL) and TBC (5.67 log10 CFU/mL) were significantly higher three weeks before kidding than five weeks after.

Taken together, the reviewed studies indicate that seasonal SCC variation reflects the combined influence of photoperiod, reproductive activity, lactation stage, environmental conditions, and herd management [53, 57, 62]. Therefore, season should be considered as an interacting physiological factor rather than an isolated determinant when interpreting SCC in dairy goats.

#### Influence of udder conformation on SCC

Udder conformation may influence SCC, although associations remain inconsistent. Some studies found no significant SCC differences between udder halves or in relation to milk composition [33, 62, 71].

Conversely, significant associations between SCC and udder morphology, including shape, symmetry, separation degree, perimeter, and depth were identified in the study [72]. SCC was highest in pendular cylindrical udders (9,058.1 × 10³ cells/mL) compared with globular (2,001.5 × 10³ cells/mL) or pear-shaped (1,585.3 × 10³ cells /mL), higher in asymmetrical (6,759.7 × 10³ cells /mL) than symmetrical udders (3,729.7 × 10³ cells /mL), and greatest in udders with severe separation (10,452.3 × 10³ cells /mL) compared with slight separation (2,051.3 × 10³ cells/mL) [72]. Similarly higher SCC and greater subclinical mastitis susceptibility were reported in cylindrical-shaped udders [73].

Negative correlations between SCC and udder depth, width, and circumference were also reported in the research [34], while negative associations with udder length and cleft were reported n the study [73]. In contrast, positive correlations between SCC and udder dimensions were observed in the research [71]. No differences related to udder length or circumference were found in another study, although higher SCC (> 6,000 × 10³ cells/mL) and TBC were reported in udders closer to the ground [14].

Teat characteristics showed similarly inconsistent results. Negative correlations between SCC and teat size were reported in study [34], whereas positive associations were described in another study [71]. Longer teats were observed in goats with SCC > 2,000 × 10³ cells/mL, while no significant correlations were found in another study [32].

Overall, although certain morphological traits may increase SCC and mastitis risk, no universally consistent relationship between udder conformation and SCC has been established [14].

#### Milking type and frequency impact on goat milk SCC and composition

Milking parameters are associated with SCC variation, though findings are inconsistent. Reduced milking frequency increased SCC and microbial load [74], whereas only slight increases and no significant effect were observed in another studies [75, 76]. Breed differences in SCC with no consistent frequency effect were observed in the study [77].

Milking type also yielded variable results. Higher SCC in machine-milked goats (0.787 × 10⁶ cells/mL) than in manually milked animals (0.350 × 10⁶ cells/mL) were reported in study [78], while higher SCC in hand-milked herds (1,121,000 cells/mL) compared with machine-milked herds (848,000 cells/mL), with milking system explaining 8% of variability were found in another study [54].

Machine settings further influence SCC. Liner diameter affected SCC and peak flow rate, whereas vacuum level (44 vs. 35 kPa) did not alter SCC but influenced other milk variables [79]. Another report [80] reported increased risk of high SCC at vacuum levels < 40 kPa, while study [81] found no SCC differences between 42 and 44 kPa despite higher teat edema at higher vacuum. Some other reports found no significant associations between milk flow characteristics and SCC [14, 82, 83].

Overall, milking frequency, system, and equipment parameters may affect SCC, but effects are context-dependent and interact with breed and management.

#### Goat farming system

Farming system is associated with SCC variability, although findings remain inconsistent. Higher SCC in an intensive herd (1,875 ± 476 × 10³ cells/mL) compared with a small-scale operation (895 ± 112 × 10³ cells/mL), indicating management influence were reported [84]. Similar research [59] showed that farm type (traditional, intensive, modern) accounted for 16% of SCC variability.

Conventional (800 × 10³ cells/mL; log SCC 6.33), traditional (735 × 10³ cells/mL; log SCC 6.69), and organic farming systems (150 × 10³ cells/mL; log SCC 5.68) were compared in the research [85]. Although no significant differences were detected, the observed SCC values varied among production systems, with the lowest mean SCC recorded in organic farms. Some researchers [48] observed only minor differences between extensive (log2 6.67) and intensive systems (log2 6.57). These findings suggest that individual herd management practices may have a greater influence on SCC than farming system classification alone.

Environmental stress represents an important non-infectious determinant of SCC. Acute stress increased plasma cortisol and significantly elevated SCC, particularly in older goats [86]. Milk production decreased by 11% on the first day after stress exposure, and lactose concentration declined, while other milk components were largely unaffected. These results indicate that stress-induced physiological responses can elevate SCC independently of infection.

In summary, although intensive systems are often associated with higher SCC, management practices, environmental stressors, and herd-specific conditions appear to be more decisive determinants of SCC levels [86].

#### Goat feeding

Nutrition plays a key role in regulating SCC by influencing immune function and udder health. Dietary cation–anion difference (DCAD) affects SCC: lower SCC in goats fed a negative DCAD diet (121.0) compared with zero (194.0) and positive DCAD (267.6) were reported, with the negative group also showing the highest milk yield [87].

Plant-based supplements have shown beneficial effects. Lower SCC in goats fed pellets containing 70% sun-dried moringa biomass were observed [88]. Reduced SCC in goats fed 100% purple Napier grass silage (1.14 × 10⁶ cells/mL) compared with 50% silage (1.26 × 10⁶) and control diets (1.93 × 10⁶) also were reported [89]. As it was reported in one study, marine algae supplementation (10 g/day) decreased SCC (5.34 vs. 5.73 log cells/mL) and reduced udder pathogen prevalence (10% vs. 31%) without impairing milk composition [90].

Herbal supplementation also influenced SCC. Lower SCC was observed in goats supplemented with *Echinacea purpurea* (451 × 10³ cells/mL) or *Echinacea* plus chamomile (475 × 10³ cells/mL) compared with the control (611 × 10³ cells/mL) and chamomile alone (505 × 10³ cells/mL) [91]. *Echinacea* additionally improved milk yield and composition, However, no substantial differences in SCC were observed (387.38–426.11 × 10³/cm³), although milk colour intensity was reduced [92].

Mineral, vitamin, marine oil, and essential oil supplementation were reviewed in the research [93]. Selenium and vitamin E combinations were more effective in sheep than goats, while polyphenols from agricultural by-products reduced SCC. Essential oils may decrease SCC but can negatively affect milk flavour. Condensed tannins (Lespedeza cuneata, 15.2%) reduced SCC through probable bactericidal effects [94]. Extruded flaxseed reduced SCC by 6.89% or showed no effect [94]. Coffee grounds silage rich in polyphenols (50 g/day) also reduced SCC in goats with subclinical mastitis [95].

Overall, feeding strategies incorporating antioxidative, anti-inflammatory, and immunomodulatory components can support udder health and contribute to SCC reduction, although responses depend on supplement type, dose, and individual variation [88–93].

#### Summary

The evidence presented in this section demonstrates that SCC in goat milk is influenced by a broad range of physiological, anatomical, environmental, and management-related factors. Unlike in other dairy species, baseline SCC values in goats are inherently higher due to the apocrine mechanism of milk secretion and the presence of cytoplasmic fragments. However, beyond this species-specific characteristic, SCC variability is strongly associated with lactation stage, parity, reproductive status, season, milk composition, udder conformation, milking practices, farming system, and feeding strategies [88–93].

While certain determinants—such as late lactation, increasing parity, estrus, and environmental stress—consistently show positive associations with SCC elevation, other factors including birth type, udder morphology, milking frequency, and production system demonstrate contradictory findings across studies. These inconsistencies suggest that SCC in goats is not governed by a single dominant factor but rather by complex interactions between physiological status and external conditions [88–93].

To facilitate systematic interpretation, the principal physiological and management-related determinants of SCC variability are summarized below in Table 1.

**Table 1:** Physiological and management-related factors influencing SCC in goat milk

Factor	Direction of SCC effect	Consistency across studies	Key consideration
Lactation stage	↑ in late lactation	Consistent	Physiological increase
Parity	↑ with increasing parity	Mostly consistent	Cumulative exposure effect
Estrus	Transient ↑	Confirmed	Hormonal influence
Season	Variable	Inconsistent	Linked to reproduction & climate
Birth type	Variable	Contradictory	Management-dependent
Udder conformation	Some traits ↑ SCC	Inconsistent	No universal correlation
Milking frequency /system	Variable	Inconsistent	Parameter-dependent
Farming system	Variable	Inconsistent	Management practices influential
Environmental stress	↑ SCC	Supported	Cortisol-mediated response
Feeding strategies	Often ↓ SCC	Moderately consistent	Anti-inflammatory /immunomodulatory effects

Overall, the summarized findings confirm that physiological and management-related factors substantially influence SCC variability in goats. Importantly, not all SCC increases are indicative of pathology. Several elevations reflect normal physiological adaptations related to lactation dynamics, hormonal changes, or environmental stress. At the same time, the contradictory results observed for certain factors emphasize the need for cautious interpretation and context-specific evaluation.

These findings reinforce the concept that SCC in goat milk cannot be treated as a static diagnostic parameter or interpreted solely through fixed threshold values. Instead, a comprehensive understanding of physiological background and production conditions is required for accurate milk quality assessment and herd health management.

While physiological and environmental determinants explain a substantial proportion of SCC variability, they do not fully account for the observed inter-individual and breed-specific differences. This suggests that intrinsic biological factors may also contribute to SCC regulation. Therefore, beyond physiological influences, it is essential to consider the role of genetic background and specific polymorphic markers in shaping SCC expression in goats [95].

The following section examines genetic and breed-related determinants of SCC variability and their potential implications for selection and breeding strategies in dairy goat production.

### Genetic factors influencing SCC in goat milk

Genetic background is considered one of the intrinsic factors influencing SCC in goats. However, the impact of breed and specific genetic polymorphisms on SCC remains inconsistent across studies, suggesting that genetic effects may interact with environmental and management conditions.

#### Breed-related genetic variation in SCC

Breed has been reported as a factor influencing milk SCC; however, findings across studies are not always consistent. For instance, significantly higher SCC was reported in Hair goats (6.01 × 10⁵ cells/mL) compared with Alpine × Hair F1 (4.92 × 10⁵ cells/mL) and Saanen × Hair F1 goats (5.29 × 10⁵ cells/mL) [58]. Similarly, variation in log SCC values (6.37–6.73) was observed among breeds, with lower values in Saanen–Maltese crosses and higher values in Sarda goats; however, these differences were not statistically significant [48]. In contrast, other findings suggested that breed may influence cheese yield primarily through milk yield and composition rather than directly affecting SCC [96, 97].

Other studies have reported limited or no significant breed-related differences. Significant differences in SCC between breeds were not detected, although substantial individual variability was noted [37, 65, 98]. Likewise, no significant SCC differences between Greek and imported breeds (0.894 × 10⁶ vs. 0.754 × 10⁶ cells/mL) were reported, although breed-related effects became evident when bulk tank SCC exceeded 0.75 × 10⁶ cells/mL [99].

Overall, breed-specific differences in SCC appear to be context-dependent and may reflect both genetic background and environmental or management influences (Table 2). Some breeds, such as Murciano-Granadina, have been characterized by relatively stable SCC levels under specific management conditions, which may indicate a combination of genetic predisposition and physiological adaptability [100]. In contrast, Malagueña goats have exhibited lower SCC compared with Florida goats under similar management conditions, suggesting possible genetic differences between breeds [101]. Nigerian Dwarf goats have been reported to present higher mean SCC values, indicating potential breed-dependent variation in SCC expression [102].

Additionally, some authors have emphasized that observed breed differences may reflect selection intensity and adaptation to production systems rather than inherent resistance or susceptibility to mastitis [103]. For example, Saanen goats have been reported to exhibit higher SCC compared with Mediterranean breeds such as Maltese, Jonica, Rossa Mediterranea, Girgentana, and Garganica [104]. These findings highlight the importance of considering breed-specific physiological baselines when interpreting SCC values in goat milk [105]. (Table 2).

**Table 2:** Breed-related differences in goat milk SCC

Breed / Comparison	SCC value	Main finding	Reference
Hair vs. Alpine × Hair F1 vs. Saanen × Hair F1	6.01 vs. 4.92 vs. 5.29 × 10⁵ cells/mL	Higher SCC in Hair goats	[58]
Saanen–Maltese vs. Sarda	log 6.37–6.73	Differences not significant	[48]
Greek vs. imported breeds	0.894 vs. 0.754 × 10⁶ cells/mL	No significant difference	[99]
Saanen vs. Mediterranean breeds	—	Higher SCC in Saanen goats	[104]
Multiple breeds (various studies)	—	No consistent breed effect	[37, 65, 98]

Additionally, some studies have emphasized that breed differences may reflect selection intensity and adaptation to production systems rather than inherent resistance or susceptibility to mastitis [103]. Saanen goats have been reported to exhibit higher SCC compared with Mediterranean breeds such as Maltese, Jonica, Rossa Mediterranea, Girgentana, and Garganica [104]. These findings indicate that breed-specific physiological baselines should be considered when interpreting SCC values [105].

#### Polymorphic genetic factors influencing SCC

SCC in dairy goats also demonstrates a measurable heritable component, indicating that part of the phenotypic variation in SCC is attributable to additive genetic effects. Genetic parameter estimates reported for Alpine and Saanen goats suggest low-to-moderate heritability of milk somatic cell score, depending on breed, parity, lactation stage, and the statistical model applied [105]. These estimates indicate that genetic selection may contribute to long-term improvement of udder health, although the relatively moderate heritability also confirms the substantial influence of environmental, physiological, and management-related factors. Genetic correlations between somatic cell score, milk production, and udder type traits further suggest that SCC should be considered within multi-trait selection indices rather than as an isolated breeding criterion.

Beyond breed-level variation, several genetic polymorphisms have been investigated in relation to SCC in goats. Four single nucleotide polymorphisms (SNPs) in the promoter region of the lactoferrin gene associated with SCC and milk production traits were identified [92]. Lower SCC values were observed in genotypes TT (SNP1), AA (SNP3), GG (SNP4), and TC (SNP2), and certain genotypes were also associated with increased milk protein and lactose content.

Polymorphisms in immune- and production-related genes have also been described. A C/T SNP in exon 4 of the growth hormone receptor (GHR) gene was associated with milk production traits and may indirectly influence SCC through effects on milk synthesis and mammary gland function [106]. Furthermore, the SNP rs650082970 in the TLR2 gene showed a significant association with SCC, and the A allele was suggested to confer increased resistance to mastitis-related inflammation [107, 108].

Breed-specific differences in SCC have been reported, which may reflect underlying genetic variation as well as differences in physiological adaptability and management conditions.

These findings support the inclusion of SCC-related genetic markers in breeding programs aimed at improving resistance to subclinical mastitis and enhancing milk quality [109–111]. Nevertheless, although SCC has a heritable component, current evidence indicates that genotype alone does not fully explain SCC variability, and environmental and management factors remain highly influential.

**Table 3:** Genetic polymorphisms associated with SCC in goats

Gene	Polymorphism / Genotype	Association with SCC	Additional effect	Reference
Lactoferrin	TT (SNP1), AA (SNP3), GG (SNP4), TC (SNP2)	Lower SCC	↑ protein / lactose (some genotypes)	[97]
GHR	C/T SNP (exon 4)	Associated with milk traits	Production traits	[106]
TLR2	rs650082970 (A allele)	Lower SCC / mastitis resistance	Immune response	[107, 108]

Overall, the available evidence indicates that specific genetic polymorphisms (Table 3), including variants in the lactoferrin, GHR, and TLR2 genes, may be associated with variation in SCC and resistance to mastitis. However, the reported effects are not consistent across breeds and production systems. Although SCC demonstrates a heritable component and may serve as a useful criterion in selection programs [109–111], genetic background alone does not fully explain SCC variability. Therefore, interpretation of SCC should consider both polymorphic genetic factors and the strong influence of environmental and management conditions.

In summary, genetic background contributes to variation in SCC in goats at both breed and molecular levels. Although certain breeds have been reported to exhibit higher or lower SCC values, these differences are not consistently confirmed across studies and often depend on management systems, production conditions, and physiological status. Substantial individual variability within breeds further complicates interpretation.

At the molecular level, polymorphisms in genes such as lactoferrin, GHR, and TLR2 have demonstrated associations with SCC and, in some cases, with resistance to mastitis or milk production traits. These findings indicate that SCC possesses a heritable component and may be considered in breeding programs aimed at improving udder health and milk quality. However, the magnitude of genetic effects appears moderate, and genotype alone does not fully explain SCC variability [106–108].

Therefore, while genetic selection may support long-term improvement of udder health, SCC expression in goats results from a complex interaction between genetic predisposition and non-genetic factors. Given that SCC reflects not only genetic background but also immune response activity and inflammatory processes within the mammary gland, it is essential to examine the role of infectious and inflammatory factors. The following section focuses on inflammatory mechanisms influencing SCC in goat milk, with particular attention to mastitis and associated diagnostic indicators.

### Inflammatory factors influencing SCC in goat milk

#### Impact of infected goat udder on SCC

Mastitis is a major health concern in dairy goats, affecting milk quality and yield. Goat udders are frequently infected with pathogens like those causing bovine mastitis. Coagulase-negative *staphylococci* (CNS) are the most common and are associated with persistent infections, elevated SCC, and subclinical mastitis with occasional clinical episodes. Although SCC is widely used as an indicator of IMI, it is also influenced by physiological factors such as parity and lactation stage, complicating interpretation [112].

IMI is one of the principal determinants of SCC. As it was reported SCC was more affected by parity and lactation stage in infected goats than in uninfected ones [57]. Mean SCC adjusted for parity and days in milk was 780,000 cells/mL in culture-negative goats and 794,000 cells/mL in culture-positive goats, compared with 522,000 cells/mL in goats without bacteriological samples. During late lactation, SCC remained significantly higher in culture-positive goats; however, even uninfected goats exceeded 1,000,000 cells/mL in advanced lactation [57].

A positive association between TBC and SCC was reported during the kidding season [62], with strong correlations between udder halves and between individual SCC and TBC values. Similar research [14] found significantly higher TBC in goats with SCC > 2,000 × 10³ cells/mL, and both parameters were positively associated with parity. As it was demonstrated in another research [55] mastitis pathogens were significantly more frequent in high-SCC groups (301–1,000 × 10³ and > 1,010 × 10³ cells/mL) than in low-SCC milk (< 300 × 10³ cells/mL), accompanied by higher PMN proportions [55]. Other reports [69, 70] confirmed significantly higher SCC in bacteriologically positive or SCC > 1,000,000 cells/mL mammary glands. In contrast, no correlation was found between SCC and TNC, despite TNC ranging from 10⁴–10⁵ CFU/mL in intensive herds and 2.7 × 10⁴–6.2 × 10⁴ CFU/mL in small farms [84].

Strong associations between bacterial contamination, SCC, and leukocyte fractions also were reported [65]. In milk with low contamination, leukocytes comprised ~ 35% of SCC, whereas in samples with major pathogens they reached ~ 50%, with increased neutrophil, monocyte, and eosinophil proportions. Nevertheless, approximately 20% of low-SCC samples (< 1 × 10⁶/mL) still contained pathogens [65]. Similar conclusions were presented also in another study [113].

As it was identified in the study [114] *Staphylococcus caprae* (6.8%), *Staphylococcus warneri* (6.3%), and *Staphylococcus epidermidis* (3.8%) are the most common udder pathogens, each contributing modestly to SCC, whereas *Staphylococcus aureus* (3.6%) had a more pronounced impact. It was also reported [112] that subclinical mastitis was mainly caused by non-staphylococci bacteria, while clinical cases involved *Enterobacteriaceae*, *Corynebacterium spp*., *Pseudomonas spp*., *Mycoplasma spp.*, and *Aspergillus fumigatus*.

IMI induce oxidative stress in udder tissue. Alterations in milk antioxidant enzymes—catalase, lactoperoxidase, glutathione peroxidase, and xanthine oxidase—may serve as indicators of subclinical mastitis [115].

According to EU legislation, although non-infectious factors influence SCC, subclinical mastitis remains the primary cause of SCC elevation (Table 4). Therefore, SCC determination and the California mastitis test (CMT) remain valuable screening tools in goats, despite lower predictive accuracy than in sheep [10, 114].

**Table 4:** Major pathogens and their association with SCC in goats

Pathogen / Condition	SCC Association	Additional Findings	Reference
Coagulase-negative *staphylococci*	Persistent ↑ SCC	Subclinical mastitis	[112]
*Staphylococcus aureus*	Significant ↑ SCC	3.6% prevalence	[114]
*Staph. caprae*,* S. warneri*,* S. epidermidis*	Moderate ↑ SCC	6.8%, 6.3%, 3.8% prevalence	[114]
High SCC groups (301–1000 × 10³; >1010 × 10³)	More frequent pathogens	↑ PMN	[55]
SCC > 1,000,000 cells/mL	Associated with bacteriological positivity	—	[63, 64]
Low SCC (< 1 × 10⁶/mL)	20% still pathogen-positive	Diagnostic limitation	[65]
TNC 10⁴–10⁵ CFU/mL	No SCC correlation	Intensive vs. small farms	[84]

#### Electrical conductivity and infected udder

Overall, IMI remains the principal cause of elevated SCC in goats, although physiological and management factors may modulate SCC expression. While high SCC generally correlates with bacterial presence, the occurrence of pathogens in low-SCC milk highlights diagnostic limitations and the need for combined bacteriological and SCC-based evaluation.

**Fig. 3:**
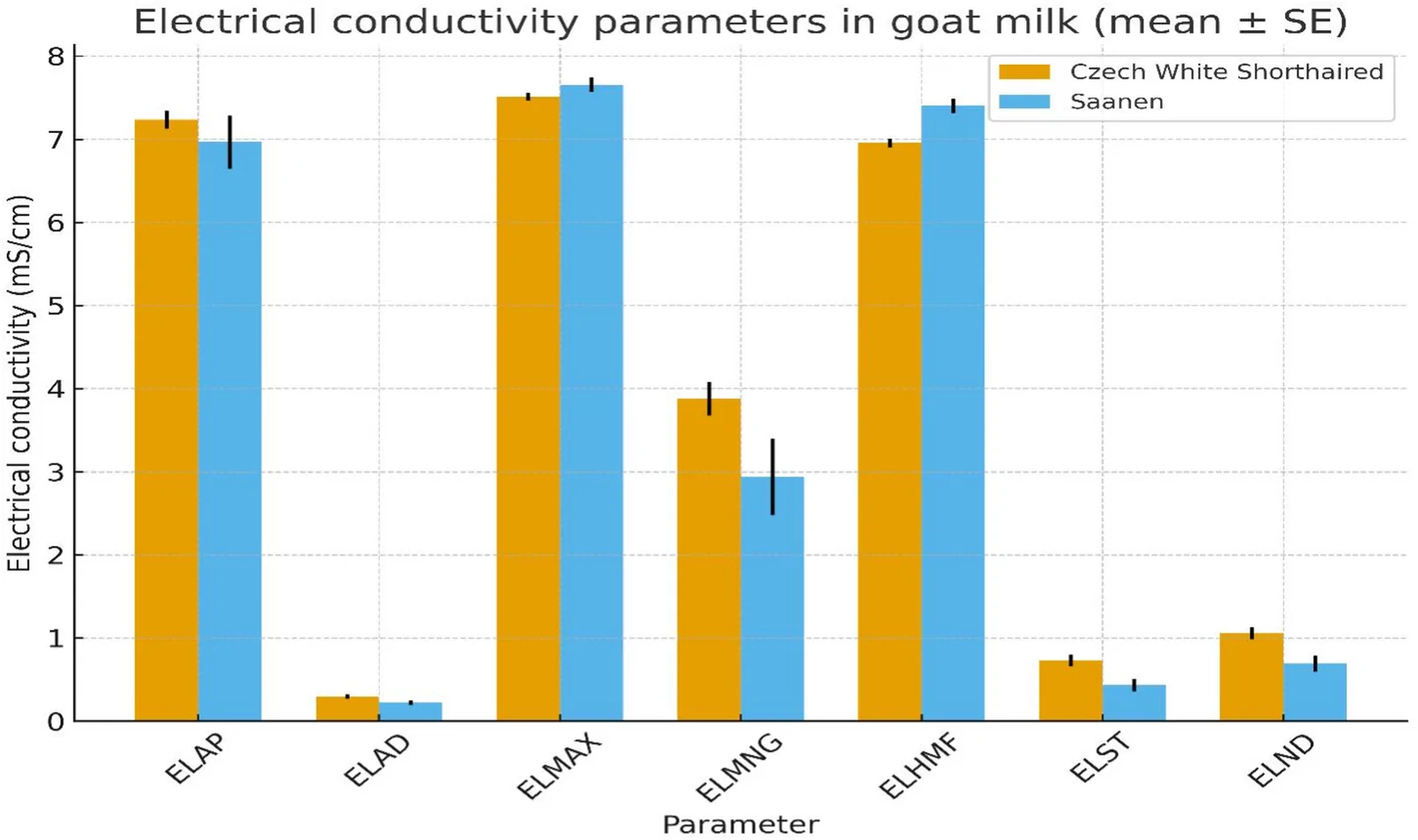
Electrical conductivity parameters in milk from Czech White Shorthaired and Saanen goats. Values are expressed as mean ± standard error (mS/cm) for ELAP (average plateau EC), ELAD (descending EC), ELMAX (maximum EC), ELMNG (mean EC), ELHMF (half milk flow EC), ELST (stability EC), and ELND (descending phase EC). Data adapted from [109]

Milk electrical conductivity (EC) is a useful indicator of udder health, reflecting alterations in milk ionic balance caused by inflammation and mammary tissue damage. Alongside SCC, EC serves as a rapid and non-invasive tool for detecting subclinical and clinical mastitis (Table 5).

EC in healthy and non-healthy glands (positive bacteriology or SCC > 2,000,000 cells/mL) were evaluated in the study [69]. Mean EC and σ²EC values were significantly higher in non-healthy glands than in healthy ones (13.69 ± 0.67 vs. 12.70 ± 0.13 mS/cm; 0.169 ± 0.012 vs. 0.098 ± 0.006 mS/cm, respectively). Both parameters were lowest during first lactation and increased with advancing lactation stage; however, no interaction between health status and lactation stage was detected [64]. Similar results were reported in additional studies [69, 70], confirming higher EC values in non-healthy glands throughout lactation.

Breed-related variability has also been described. As shown in Fig. 3, Czech White Shorthaired and Saanen goats exhibited distinct EC profiles, particularly in ELMAX, ELHMF, and ELST parameters, indicating a possible genetic influence on EC variability [109].

EC in healthy glands, bacterial mastitis, and unspecified mastitis (negative bacteriology with SCC > 1,000,000 cells/mL) were compared in research [60]. EC was highest in unspecified mastitis (5.41 and 5.95 mS/cm in farms 1 and 2) and lowest in bacterial mastitis (5.09, 5.69, and 5.73 mS/cm in farms 1, 2, and 3). In primiparous goats, EC in unspecified mastitis was significantly higher than in healthy or bacterial mastitis glands, whereas multiparous goats exhibited higher EC overall. EC increased with lactation stage in multiparous goats but not in primiparous animals. A moderate positive correlation between EC and SCC was observed, strongest in glands with unspecified mastitis (SCC > 1,000,000 cells/mL) [60].

Automated EC measurement in Murciano-Granadina goats using 19 algorithms based on gland EC and RATEC (ratio of gland EC to cisternal EC) were assessed in the research [116]. All clinical cases (*n* = 2) were detected (100% sensitivity). Global sensitivity for clinical and subclinical mastitis ranged from 33.3 to 58.3%, and specificity from 77.8 to 100%, comparable to results reported in cows using ARIMA and Rule 1 algorithms. The most severe cases were detected rapidly with low false-positive rates [116].

**Table 5:** Diagnostic performance of electrical conductivity (EC) for mastitis detection in goats

Study	Health status definition	Main EC findings	Diagnostic performance	Reference
Zaninelli et al.	Positive bacteriology or SCC > 2,000,000 cells/mL	Higher EC and σ²EC in non-healthy glands	—	[69]
Zaninelli et al.	Non-healthy vs. healthy glands across lactation	EC increased with lactation; higher in infected glands	—	[69, 70]
Diaz et al.	Bacterial mastitis; unspecified mastitis (SCC > 1,000,000 cells/mL)	Highest EC in unspecified mastitis; moderate EC–SCC correlation	—	[60]
Romero et al.	Intramammary infection (clinical & subclinical)	Automated EC and RATEC evaluation	SENS 33.3–58.3%; SPEC 77.8–100%; 100% detection of clinical cases	[116]
EC screening studies	Subclinical mastitis	Specificity rarely > 95%; high false positives; fuzzy logic reduces FP	Improved diagnostic accuracy with new algorithms	[60, 116, 117]

However, the effectiveness of EC for subclinical mastitis detection remains variable. Specificity rarely exceeds 95%, and with low disease prevalence this results in a high number of false positives. Consequently, early treatment decisions based solely on EC are limited in practice. The application of new algorithms, including fuzzy logic approaches, has demonstrated a significant reduction in false positives and may improve the feasibility of EC-based early detection systems [60, 116, 117].

#### Goat milk composition and SCC tracking methods

Goat milk composition is evaluated based on key parameters such as fat, protein, lactose, mineral content, and SCC, which reflects milk quality and udder health. SCC monitoring methods include traditional microscopic counting, flow cytometry, automated electronic counters, and more recently, machine learning algorithms. Specialized machine learning tools adapted for small ruminant farms was described in the study [118]. Their findings indicate that algorithms such as decision trees, random forests, XGBoost, k-nearest neighbors, and neural networks can be used to predict milk quality. Computational models developed using field data demonstrated strong performance for parameters related to milk production, including protein content (sheep and goats), fat and protein (sheep), SCC (sheep and goats), and total bacterial counts (goats). These models can support clinical decision-making and herd management aimed at improving milk quality [118].

While earlier work focused mainly on classification and prediction of subclinical mastitis, the later study addressed continuous target variables, enabling prediction of five quality-related parameters [118]. Machine learning approaches for small ruminant farms were further refined. The objective was a comprehensive evaluation of milk hygiene status and cheese production potential in large-scale sheep and goat farms [118].

Methods for determining SCC in goat milk using instrumental techniques (“Somatos” and “SomaCount Flow Cytometer”) and direct microscopic counting with different staining methods (May-Grünwald, Romanovsky-Giemsa, Pyronin Y) were compared in the study [119]. Direct microscopic counting in stained films was the most accurate but more time-consuming. Among staining methods, May-Grünwald proved the most effective and economical, providing clear visualization of cytoplasm and nuclei at a reagent cost 28.4 times lower than Pyronin Y. An increase in milk fat content was observed alongside higher SCC levels, confirming the influence of SCC on milk composition and its relevance as a quality indicator [119].

## Concluding remarks

The findings of this review highlight a fundamental challenge in the interpretation of SCC in goat milk: its biological meaning is not fixed but strongly context-dependent. Unlike in cattle, where SCC is more directly associated with IMI, in goats it reflects a combination of physiological, environmental, and pathological processes [6, 9, 26]. This inherent complexity limits the reliability of SCC as a standalone diagnostic indicator.

A consistent observation across studies is the substantial variability in SCC values and their inconsistent association with udder health status [14, 57, 62]. This inconsistency may be explained by differences in study design, including variation in breeds, lactation stages, management systems, and analytical methods used for SCC determination [48, 58, 65]. In particular, the influence of physiological factors such as late lactation and parity may mask or mimic pathological changes, leading to potential overestimation of mastitis prevalence when fixed thresholds are applied [59, 61, 62].

The reviewed evidence also indicates that genetic effects on SCC are present but moderate and highly dependent on environmental and management conditions [65, 97, 106]. This interaction may explain the contradictory findings reported for breed-related differences and polymorphisms [37, 98, 99]. Rather than acting as primary determinants, genetic factors appear to modulate baseline SCC levels, reinforcing the need to interpret SCC within a broader biological context.

Importantly, while IMI remains the primary driver of pathological SCC elevation, elevated SCC values do not uniformly indicate disease [57, 112]. This distinction is critical for both diagnostic decision-making and milk quality assessment, as misinterpretation may lead to unnecessary interventions or inaccurate evaluation of milk suitability for processing [10, 114].

**Fig. 4:**
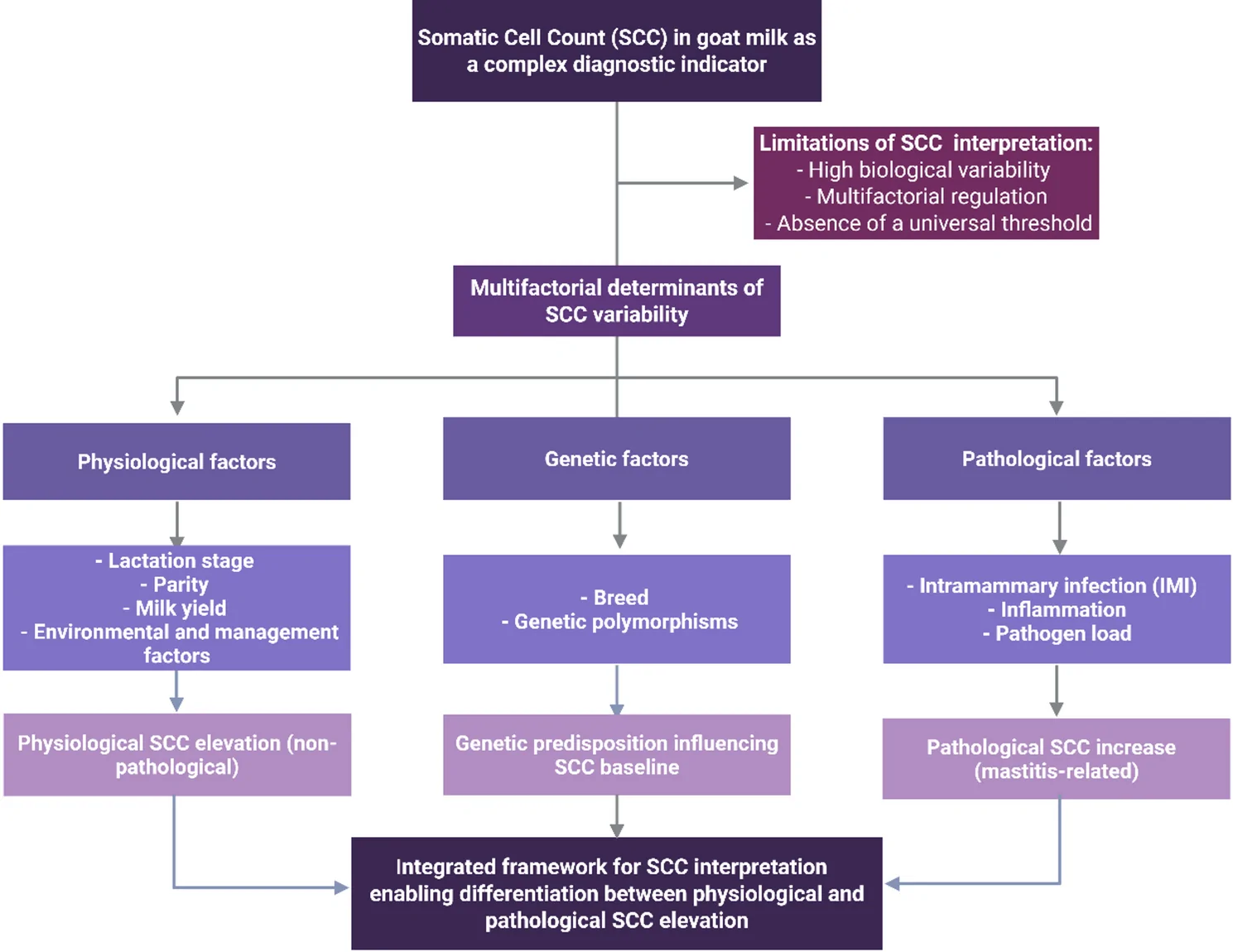
Proposed integrated framework for the interpretation of somatic cell count (SCC) in goat milk. (figure created using BioRender)

Taken together, these findings support a shift from threshold-based interpretation toward a context-dependent, integrative approach. The proposed framework (Fig. 4) synthesizes the interaction between physiological, genetic, and pathological determinants and provides a structured basis for distinguishing physiological from pathological SCC elevation [6, 9, 65]. This approach addresses a key limitation identified in the literature and contributes to a more accurate and biologically meaningful interpretation of SCC in goat milk.

Finally, the heterogeneity of the reviewed studies limits direct comparability and may partially account for inconsistencies in reported effects [14, 62, 65]. Differences in experimental conditions, definitions of subclinical mastitis, and SCC measurement techniques highlight the need for greater methodological standardization. Future research should therefore focus on harmonized study designs and context-specific diagnostic criteria to improve the reliability and applicability of SCC-based assessment in goat production systems [10, 112].

The model illustrates the interaction between physiological, genetic, and pathological determinants of SCC variability and highlights the importance of context-dependent evaluation in distinguishing physiological from pathological SCC elevation.

By resuming it could be said that the available evidence indicates that SCC variability in goat milk results from complex interactions among physiological, infectious, genetic, environmental, and management factors, indicating that a single SCC threshold is insufficient for accurate interpretation. Instead, a holistic interpretation framework integrating physiological context, infection status, genetic background, and advanced monitoring approaches is recommended to support regulatory decision-making, improve udder health assessment, and optimize milk processing outcomes. This review synthesizes the current evidence while acknowledging the considerable heterogeneity among studies, highlighting the need for standardized methodologies and further research to refine SCC interpretation in dairy goats.

## Data Availability

No datasets were generated or analysed during the current study.

## Declarations

### Prior publication

Data have not been published previously.
